# Economic evaluation of evidence-based strategies to reduce unhealthy alcohol use: a resource allocation guide

**DOI:** 10.3389/fpubh.2025.1466552

**Published:** 2025-08-11

**Authors:** Delia Hendrie, Ted R. Miller

**Affiliations:** ^1^School of Population Health, Curtin University, Perth, WA, Australia; ^2^Pacific Institute for Research and Evaluation, Beltsville, MD, United States

**Keywords:** alcohol harm, alcohol policy, prevention, resource allocation, economic evaluation

## Abstract

**Introduction:**

In the context of limited budgets to implement public health measures, cost-effectiveness is an important factor for policy makers to consider. Evidence from high-income countries on the outcomes and costs of interventions demonstrating success in reducing alcohol-related harm offers valuable guidance for resource allocation decisions in low- and middle-income settings.

**Methods:**

Published reviews of interventions shown or likely to reduce alcohol-attributable harm were identified. Data on outcomes was extracted and standardized to allow consistent reporting of return on investment. Intervention costs were calculated using a ‘bottom-up’ approach based on quantity of resources and unit price. Benefit–cost ratios and incremental cost-effectiveness ratios were calculated for each intervention.

**Results:**

Across the various categories of alcohol harm reduction programs, interventions demonstrating good value for money were identified. These categories were availability of alcohol; marketing of alcoholic beverages; pricing policies; drink driving policies; workplace interventions; health sector programs; youth development programs; and school-based substance abuse prevention.

**Conclusion:**

Consistent estimates of return on investment of alcohol harm reduction interventions provide an effective mechanism to filter out interventions of questionable value. Policymakers should also consider factors such as political feasibility, local priorities, cultural appropriateness, affordability, and the immediacy of impact when selecting a comprehensive package of strategies.

## 1 Introduction

Harmful alcohol consumption is an important public health problem. Globally alcohol consumption was responsible for 4.6% of total disability-adjusted life years (DALYs) in 2019 (1), with alcohol a risk factor for over 200 conditions including cardiovascular diseases, cancers, mental and neurological disorders as well as transport injuries (2). Beyond health consequences, the harmful use of alcohol is associated with significant social and economic losses to individuals and society (3, 4).

Although rates of age-standardized DALYs from alcohol use are lower in low- and middle- income (LMIC) settings than high income countries, these countries accounted for 76% of alcohol-attributable deaths in 2021 (5). With alcohol consumption reducing in many high-income countries, the global alcohol industry has increasingly turned its focus toward emerging markets where regulations to control alcohol use are less stringent (6). In these regions, alcohol companies employ campaigns and tactics like those used in wealthier countries, combining global themes with local cultural icons and celebrations to attract consumers (7). Additionally, some multinational alcohol companies form partnerships with global health organizations, presenting themselves as socially responsible, yet often without reducing alcohol availability or consumption (8). This poses a challenge to the global goal of reducing the harmful effects of alcohol as expressed by the WHO Global Alcohol Action Plan 2022–2030 (9). Reducing alcohol consumption is also highly relevant to achieving many of the health-related targets of the Sustainable Development Goals (1).

Effective alcohol policy measures exist that can significantly reduce alcohol consumption and mitigate alcohol-related harm. However, limited literature is available focusing specifically on LMIC countries. Only two recent studies have reviewed literature on effectiveness of interventions to reduce harmful alcohol use, specifically in LMIC countries. A systematic review by Staton et al. assessed the effectiveness of patient-level interventions, finding that brief interventions were the most commonly studied and consistently showed positive results (10). The second study, a Cochrane review, focused on psychosocial and pharmacological treatments for reducing harmful alcohol use in LMICs. Certainty of evidence on effectiveness was found to be low (11). These reviews suggest limited strong evidence is available on the effectiveness of alcohol harm reduction strategies in LMICs (12); even less is available on the cost-effectiveness of policies.

Given the limited budgets available for public health measures, cost-effectiveness becomes an important factor for policy makers to consider in decisions about allocating scarce resources to prevent excessive alcohol consumption. Evidence from high-income countries demonstrates that interventions such as implementing pricing and taxation policies, regulating alcohol sales hours and outlet density, and enforcing drink-driving laws can be successful in reducing alcohol-related harm (13, 14). While contextual factors are likely to impact on the transferability of findings on return on investment from high- to LMIC countries (15), an evidence-based list of policy options, together with applying a logical reasoning approach for each country, can be helpful in developing a portfolio of policy options that are evidence-based and likely to be cost-beneficial in these settings (12).

This study builds on existing literature about successful initiatives that have reduced alcohol-related harm, producing comparable cost-outcome estimates localized to the Western Australian context, a jurisdiction in a high-income country. These estimates can serve as a reference for LMIC countries, helping to identify interventions that offer good value for money when adapted to local contexts and cultural considerations. Furthermore, using benefit transfer methods, cost-outcome measures from this study can be adjusted and applied, with caution, to generate return on investment estimates in the context of other settings (16).

## 2 Methodology

This section outlines the methodology adopted in producing cost-outcome measures of interventions to reduce alcohol-attributable harm. The first task was to produce a “What Works?” list of proven interventions. For these interventions, economic evaluations were conducted to assess cost-effectiveness.

The remainder of this section describes the methods used, including how interventions were selected, adjustments made to some findings to ensure consistency, and steps taken in generating return on investment ratios.

### 2.1 Methods

#### 2.1.1 Identifying interventions

The approach adopted was to systematically identify published reviews and meta-analyses to find interventions shown or likely to reduce alcohol-attributable harm. The search was conducted in five stages.

1 Databases of policy interventions found to achieve improvements in outcomes were searched using ‘alcohol’ as the main search term. These included the following:

- Washington State Institute for Public Policy’s database (17).
- Substance Abuse and Mental Health Services Administration (SAMHSA)’s National Registry of Evidence-based Programs and Practices (NREPP) (18).
- The Community Preventive Services Task Force Guide to Community Preventive Services (19).
- Cochrane Database of Systematic Reviews (20).
- Joanna Briggs Institute (JBI) Database of Systematic Reviews and Implementation Reports (21).
- European Monitoring Center for Drugs and Drug Abuse (22).

2 A previous report commissioned by SAMHSA’s Center for Substance Abuse prevention that analyzed costs and benefits of substance abuse and related prevention programs was reviewed to identify additional relevant interventions (13).
3 A key researcher in alcohol policy and harm reduction in Australia was consulted and a subsequent scan of Australian government reports and journal articles conducted.
4 Searches using key terms were initially conducted in 2016 on Web of Science, PubMed, Proquest, EBSCO and Google Scholar for meta-analyses or systematic reviews to identify any relevant studies not included in steps (1) to (3) above. The following combinations of key terms were used:

- (“alcohol price” OR “alcohol tax” OR “alcohol cost” OR “drinking cost”) AND (“systematic review” or “meta-analysis”).
- (“drinking age law” OR “minimum drinking age” OR “legal drinking age”) AND (“systematic review” or “meta-analysis”).
- (“drinking age law” OR “minimum drinking age” OR “legal drinking age”) AND (“systematic review” or “meta-analysis”).
- (“random breath test” OR “sobriety checkpoint” OR “blood alcohol concentration” OR “BAC” OR “interlock program”) AND (“systematic review” or “meta-analysis”).
- (“electronic screening” or “brief intervention”) AND (“alcohol” OR “substance abuse”) AND (“systematic review” OR “meta-analysis”) AND (“prevention”)
- (“hours of sale” OR “days of sale” OR “trading hours”) AND (“alcohol”) AND (“systematic review” or “meta-analysis”)
- (“liquor license” OR “outlet density” OR “drinking venues”) AND (“systematic review” or “meta-analysis”)
- (“sales to minor” OR “underage drinking”) AND (“systematic review” OR “meta-analysis”)

5 Searches in the abovementioned databases were conducted in 2020 for any updates of evaluations of interventions identified in (1) to (4). Key terms used in the searches were (“name of intervention”) AND (“randomized control trial” OR “evaluation” OR “effectiveness”).

Criteria for interventions to be included for economic evaluation were:

- *Participants*: General population of adults or young adults.
- *Interventions*: Objective to reduce alcohol-attributable harm.
- *Outcome measures*: Must present effect size or effectiveness.
- *Types of studies*: Randomized controlled trials, interrupted time series analyses, cross-sectional time series or other designs including a credible comparison group.

Exclusion criteria were:

- Interventions targeting specific sub-groups of the population such as adults suffering from alcoholic liver disease or alcohol use disorder.
- Initiatives leading to health promotion outcomes such as an increase in knowledge or awareness of problematic alcohol consumption but not to measures that can be directly related to harm reduction.

In addition to the above criteria, interventions had to have been found to be statistically significant in reducing alcohol-related harm.

Overall, 49 interventions were included in the cost-effectiveness analysis. Broad categories of interventions included environmental interventions targeting the availability of alcohol, the marketing of alcoholic beverages, pricing policies and drink driving policies and countermeasures; workplace interventions; health sector programs; and community-based programs including youth development programs and school-based substance abuse prevention programs (Table 1).

**Table 1:** Overview of alcohol harm reduction interventions included in cost-effectiveness analysis.

Category of intervention	Type of intervention	No.
Environmental interventions	Availability of alcohol	7
	Marketing of alcoholic beverages	2
	Pricing and tax policies	4
	Drink driving policies and countermeasures	13
	Subtotal: Environmental interventions	26
Workplace interventions	Various employee groups	4
	Subtotal: Workplace interventions	4
Health sector programs	Brief alcohol interventions	3
	Subtotal: Brief alcohol interventions	3
Community-based programs	Youth development programs	8
	School based substance abuse prevention programs	8
	Subtotal: Community-based interventions	16
Total	Total: All interventions	49

Studies included in the cost-effectiveness analysis were assessed based on their quality to provide an indication of the confidence that could be attributed to their results (Table 2). The rating adopted was based on the approach adopted by Miller and Levy (23). “A” ratings were assigned to randomized controlled trials without serious attrition problems and to interventions that were implemented at a large scale and evaluated with randomized controlled trials, interrupted time series analyses, cross-sectional time series or other designs that included a credible comparison group. Rating levels declined as the quality of the evidence of effectiveness declined, based largely on the design hierarchy and review criteria in Zaza et al. including measurement bias, analytic bias and attrition bias (24).

**Table 2:** Reviewed studies, intervention descriptions and quality rating of evidence.

Category and type of alcohol harm prevention		Author/year	Intervention description	Rating
Environmental	Availability	Miller and Levy 2000 (23)	Enforcing laws against serving intoxicated patrons	B
		Miller 2001 (59); Kypri et al. 2006 (60)	Raising minimum legal drinking age to 19 yrs	A
		Miller 2001 (59)	Mandatory server training to deny service to intoxicated and underage patrons	C
		Elder et al. 2007 (61)	Enforce underage drinking laws	C
		Campbell 2009 (62); Norstrom 2010 (63)	10% alcohol outlet density reduction	B
		Hahn et al. 2010 (64); Middleton 2010 (65)	10 fewer sales hours/week	B
		Rammohan et al. 2011 (66)	Licensed establishment liability for harm caused by over-servicing patrons	B
	Marketing	Cobiac et al. 2009 (67)	Alcohol advertising ban	B
		Norstrom et al. 2010 (63)	TV alcohol advertising ban	C
	Pricing	Meng 2013 (68)	Minimum price for alcohol of A$0.95 per drink	C
		Byrnes 2012 (46)	Same tax per liter of ethanol on all alcohol, no change in total liters sold	B
		Byrnes 2012 (46)	Same tax per liter of ethanol on all alcohol, no change in total tax revenue	B
		Byrnes 2012 (46)	Spirits tax per liter of ethanol on all alcohol	B
		Byrnes 2010 (45)	Equal tax rate for beer and wine, higher rate for spirits & premised drinks	A
	Drink driving	Miller and Levy 2000 (59)	Administrative license revocation	A
		Miller and Levy 2000 (59)	Law to allow administrative license revocation based on breath testing	A
		Miller and Levy 2000 (59)	Alcohol testing ignition interlock permitted	A
		Miller 2001 (59)	Intensive random breath testing	A
		Miller 2001 (59)	Zero alcohol tolerance for drivers under 18 yrs	A
		Elder 2004 (69)	Mass media campaign to reduce drink driving	B
		Fell 2008 (70)	Saturation patrols plus media campaign	C
		Miller and Hendrie 2013 (42)	Vehicle impoundment for drink driving offenses	A
		Miller and Hendrie 2013 (42)	Electronic house arrest for drink driving offenses	A
		Miller and Hendrie 2013 (42)	Intensive probation for drink driving offenses plus treatment	A
		WSIPP 2017 (17)	Driving under the influence court	A
		Teoh 2018 (71)	Alcohol testing ignition interlock mandated for all offenders	B
		Miller 2020 (72)	Subsidized Ridesharing	B
Workplace	Various	Cook et al. 2003 (73)	Prime for Life employer-sponsored web-based health promotion program that includes substance abuse prevention	B
		Bennett et al. 2004 (74)	Team Awareness program to promote group cohesiveness, stress coping, and peer support that includes substance abuse prevention (retail and restaurant workers)	B
		Bennett et al. 2010 (75)	Team Resilience: an adaptation of Team Awareness for workers under age 26 years	B
		Spicer and Miller 2016 (76)	PREVENT: a facilitated set of employee discussions in small groups focused on recognizing the need for and planning changes in substance use and financial management for young workers	B
Health sector	Brief interventions	WSIPP 2017 (17)	Screening for heavy drinking in primary care setting and brief motivational intervention when indicated	A
		WSIPP 2017 (17)	Screening for heavy drinking in hospital inpatient setting and brief motivational intervention when indicated	A
		WSIPP 2017 (17)	Screening for heavy drinking in emergency department care setting and brief motivational intervention when indicated	A
Community	Youth development	Aos 2004 et al. (28)	Adolescent Transitions: a parenting skills program combined with universal, indicated, and selective prevention, ages 10–18	A
		Hansen et al. 2004 (77)	Across Ages: a program to strengthen adult and youth bonds through mentoring, community service and family activities, ages 9–13	A
		Hansen et al. 2004 (77)	Social Competence Promotion: a program to enhance social competence to reduce the use of violence and improve conflict resolution, ages 11–14	C
		Kuklinski et al. 2015 (78)	Communities That Care: needs assessment, followed by implementation of evidence-based youth interventions	B
		Spoth et al. 2002 (79); WSIPP 2017 (17)	Strengthening Families Program, parent–child behavioral training designed to prevent substance use, ages 12–13	A
		WSIPP 2017 (17)	Caring School Community: program to build sense of school community, ages 9–13	C
		WSIPP 2017 (17)	Good Behavior Game, classroom management strategy to teach youth to comply with rules, ages 6–9	B
		WSIPP 2017 (17)	Guiding Good Choices (Preparing for Drugfree Years), parent–child behavioral training, ages 12–13	A
	School-based	Aos et al. 2004 (28)	Family Matters: a family-focused program to reduce tobacco and alcohol use, ages 12–14	B
		WSIPP 2017 (17)	All Stars: decision-making, goal setting, and peer pressure resistance skills training, ages 11–14	B
		WSIPP 2017 (17)	Life Skills Training, 3-year program, ages 13–16	A
		Beets 2009 (80); WSIPP 2017 (17)	Positive Action: a school-wide positive behavior program aimed at improving social and emotional learning and school climate, ages 8–14	A
		WSIPP 2017 (17)	Project Northland: school-based child–parent training, 3-year program, ages 12–15	B
		WSIPP 2017 (17)	Project STAR: a school/family/community/media to prevent drug and alcohol use, 2-year program, ages 12–15	B
		WSIPP 2017 (17)	Project Toward No Drugs, ages 15–19	B
		WSIPP 2017 (17)	Too Good for drugs	B

Some reviews from which interventions were identified had already adjusted findings to increase cross-study consistency and facilitate comparison. Further adjustments were made. Table 3 classifies the adjustments in four categories: (i) recomputing cost savings with uniformly computed benefit estimates; these estimates use the effectiveness from the original studies but the unit costs of alcohol harms from previous work in Western Australia (Supplementary data), (ii) other modifications besides substituting uniform benefit estimates (e.g., recomputing program costs to capture omitted elements such as teacher time, switching discount rates or values of travel time to uniform values, or updating injury incidence rates); teacher time calculations for school-based prevention programs discussed in Miller and Hendrie (13). To match commonly used estimates, travel time was valued at 60% of the wage rate and delay time at 90% (25). (iii) computing intervention costs and cost-outcome measures as original studies provided only effectiveness estimates or incompletely computed costs, and (iv) reducing benefits by 25% when the underlying effectiveness estimates were for a demonstration stage of development because effectiveness is generally reduced when scaling up and replicating (23, 26–29).

**Table 3:** Adjustments to reviewed studies to make estimates more consistent.

Category and type of alcohol harm prevention		First author/year	Adjustments to findings			
			Cost savings	Other modifications	Intervention costs and cost-outcome measures	Demonstration stage
Environmental	Availability	Miller and Levy 2000 (23)	-	-	-	Yes
		Miller 2001 (59); Kypri et al. 2006 (60)	Yes	-	-	-
		Miller 2001 (59)	No	Yes	-	Yes
		Elder et al. 2007 (61)	-	-	Yes	-
		Campbell 2009 (62); Norstrom 2010 (63)	-	-	Yes	-
		Hahn et al. 2010 (64); Middleton 2010 (65)	-	-	Yes	-
		Rammohan et al. 2011 (66)	-	-	Yes	-
	Marketing	Cobiac et al. 2009 (67)	Yes	Yes	-	-
		Norstrom et al. 2010 (63)	-	-	Yes	-
	Pricing	Meng 2013 (68)	Yes	Yes	-	-
		Byrnes 2012 (46)	Yes	-	Yes	-
		Byrnes 2012 (46)	Yes	-	Yes	-
		Byrnes 2012 (46)	Yes	-	Yes	-
		Byrnes 2010 (45)	Yes	-	Yes	-
	Drink driving	Miller and Levy 2000 (59)	-	-	-	-
		Miller and Levy 2000 (59)	-	-	-	-
		Miller and Levy 2000 (59)	Yes	-	-	-
		Miller 2001 (59)	-	-	-	-
		Miller 2001 (59)	-	-	-	-
		Elder 2004 (69)	-	-	Yes	-
		Fell 2008 (70)	-	-	Yes	-
		Miller and Hendrie 2013 (42)	-	-	-	-
		Miller and Hendrie 2013 (42)	-	-	-	-
		Miller and Hendrie 2013 (42)	-	-	-	-
		WSIPP 2017 (17)	Yes	-	-	-
		Teoh 2018 (71)	-	-	Yes	-
		Miller 2020 (72)	-	-	Yes	-
Workplace	Various	Cook et al. 2003 (73)	-	-	Yes	-
		Bennett et al. 2004 (74)	-	-	Yes	-
		Bennett et al. 2010 (75)	-	-	Yes	-
		Spicer and Miller 2016 (76)	-	-	Yes	-
Health sector	Brief interventions	WSIPP 2017 (17)	Yes	-	-	-
		WSIPP 2017 (17)	Yes	-	-	-
		WSIPP 2017 (17)	Yes	-	-	-
Community	Youth development	Aos 2004 et al. (28)	–	–	Yes	–
		Hansen et al. 2004 (77)	–	–	Yes	–
		Hansen et al. 2004 (77)	–	–	Yes	–
		Kuklinski et al. 2015 (78)	Yes	–	–	Yes
		Spoth et al. 2002 (79); WSIPP 2017 (17)	Yes	Yes	–	Yes
		WSIPP 2017 (17)	–	–	Yes	–
		WSIPP 2017 (17)	–	–	Yes	–
		WSIPP 2017 (17)	Yes	Yes	-	Yes
	School-based	Aos et al. 2004 (28)	–	–	Yes	Yes
		WSIPP 2017 (17)	–	–	Yes	Yes
		WSIPP 2017 (17)	Yes	Yes	–	Yes
		Beets 2009 (80); WSIPP 2017 (17)	Yes	–	–	Yes
		WSIPP 2017 (17)	Yes	Yes	–	Yes
		WSIPP 2017 (17)	Yes	Yes	–	Yes
		WSIPP 2017 (17)	–	–	Yes	Yes
		WSIPP 2017 (17)	–	–	–	–

### 2.2 Data extraction

Data extracted from the reviewed studies included: (i) source of the evaluation (ii) years which the review covered (iii) intervention characteristics (iv) target population (v) details on intervention costs (if available) (vi) effectiveness measure(s) and if adjustments made (vii) follow up period of effectiveness measure(s) (viii) number of trials included in review and (ix) statistical significance.

### 2.3 Methods for assessing the cost-effectiveness of interventions

#### 2.3.1 Types of economic evaluation

Cost-effectiveness analyses of the interventions selected for inclusion comprised both cost–benefit analysis and cost-utility analysis. Cost–benefit analysis expresses the benefits of harm reduction generated by the interventions in monetary terms; cost-utility analysis expresses the benefits in quality adjusted life years (QALYs). The quality adjusted life year (QALY) is a generic measure of health, with one QALY equating to 1 year in perfect health (30).

The formula for each type of economic evaluation is as follows:

i  *Cost–benefit analysis*: Calculated as the monetary benefits from harm reduction including both lower resource costs and the monetised value of any health improvement divided by the costs of implementing the intervention. The result is expressed as a benefit–cost ratio (BCR).

$\mathrm{BCR}=\frac{\begin{array}{l} \text{reduction in resource costs}+\\ \text{monetized value of health improvement} \end{array}}{\text{costs of implementing the intervention}}$

ii  *Cost-utility analysis*: Calculated as net cost per QALY with net cost equal to the costs of implementing the intervention minus the cost savings generated from the lower resource use arising from the harm reduction divided by the value of health improvement measured in QALYs. This ratio is generally referred to as an incremental cost-effectiveness ratio (ICER) (30).

$\text{ICER}=\frac{\begin{array}{l} \text{costs of implementing the intervention}-\\ \text{reduction in resource costs} \end{array}}{\begin{array}{l} \text{value of health improvement}\;\\ \text{expressed in QALYs} \end{array}}$ .

#### 2.3.2 Approaches adopted in measuring the benefits of alcohol harm reduction interventions

In measuring the benefits of included interventions, two main approaches were adopted.

i  Intervention-specific effectiveness measures, adjusted if necessary, were obtained from the reviewed studies. These effectiveness measures were combined with estimates of (a) per person or (b) per event costs of alcohol-attributable harm (Supplementary Table), and the current incidence of the relevant harmful behavior, to determine the monetary benefits from harm reduction. Per person or per event costs of alcohol-attributable harm included the cost of both lower resource use and the monetized value of any health improvement.

The value of health improvement expressed in QALYs was then calculated by dividing the intangible cost savings generated by the alcohol harm reduction interventions by the estimate of the value of a statistical life year as recommended by the Office of Impact Analysis in the Department of the Prime Minister and Cabinet (31).

ii  Benefits of the alcohol harm reduction interventions, lower resource costs and the monetized value of any health improvement, were first calculated for the United States as most reviewed interventions were conducted in that jurisdiction. Results were then modeled to make them transferable to the Western Australian context by multiplying the United States’ benefit values by the ratio of costs per alcohol-attributable harm (per person or per event) in Australia (13, 32, 33) compared with the United States. These multipliers accounted for the differences between countries in incidence and cost per incident as well as conversion of costs between countries.

For both approaches, if interventions were effective in reducing alcohol-attributable harm, additional benefits regarding a reduction in drug use, smoking or violence were included in the benefit estimates.

#### 2.3.3 Intervention costs

Calculation of the costs of alcohol harm reduction interventions followed standard practices in cost analysis as stipulated in guidelines on the conduct of cost-effectiveness analysis (34, 35). It takes an ‘ingredients’ or ‘bottom-up’ approach, which calculates the cost of inputs as the product of the quantities used and the value (or price) of each unit (36). Data on resource use was obtained directly from the included studies or ‘best’ estimates made from other sources, including from evaluations of similar interventions reporting resource use, previous economic evaluations of alcohol harm reduction interventions, and expert opinion. The perspective taken was a partial societal one with all resource use associated with delivering the intervention included but participant costs, such as travel costs (if applicable) and time, were excluded. Resource use included time in developing and delivering the intervention, training costs if applicable, materials and equipment. Resource use that went over several years (e.g., development costs, equipment costs) were annualized to provide an annual equivalent cost and apportioned appropriately. Overhead or joint costs were excluded as the delivery of individual interventions not likely to impact to any significant extent on these costs. Total costs of interventions were calculated as the product of the quantities of all resource use consumed and the respective unit costs (or price) of resources.

Intervention costs were initially calculated for the United States and converted to the Australian context based on appropriate unit cost multipliers. Labor-oriented interventions were adjusted using the ratio of national average salaries for the relevant occupation (e.g., teacher, police officer, physician, judge, bartender/server, all employees) (37–40). Costs for interventions like ignition interlocks and media campaigns that were not labor-intensive were converted using a purchasing price parity adjuster (41).

Calculating costs of environmental interventions was more complex and varied by intervention type. Following Miller and Hendrie (42), interventions that reduced alcohol consumption—alcohol advertising bans, outlet density reduction, limits of days and hours of sales and minimum pricing policies—were costed at half the purchase price of the alcohol not consumed. This reflected the profits across the supply chain from manufacture to distribution to sales of alcohol (Miller Brewing Company), which includes the welfare loss from interventions targeting the reduction of alcohol-related harm. For these interventions, the multiplier used to adjust between countries was the ratio of before-tax drink prices (43, 44).

Currently, alcoholic beverages in Australia are subject to sales tax. Wine is taxed at a percentage of its wholesale price, while beer and spirits pay excise taxes and taxed at quite different rates with beer rates varying by container size and alcohol content. The tax changes assessed all involve moving toward a volumetric taxation system where the tax on different beverages is determined strictly by their alcohol content. Costs of those changes were computed by summing the costs of shifting to a volumetric system (45) and the “deadweight loss,” which is defined as the loss of consumer benefits resulting from the consumption decrease minus the increase in tax revenue (45, 46).

In the case of licensed establishment liability for harm caused by over-serving patrons, the estimate of intervention costs is an upper bound. It builds on data showing 51% of those stopped late-night for drink driving in Perth in 2012 got their last drink at a licensed establishment (47). This percentage was applied WA-wide and an assumption made that at most half of those who could sue licensed establishments would. The lawsuits themselves simply transfer the cost of the incident between payers, with some modest court costs added. The primary cost is to pass and implement the law. Because a cadre of licensed establishments with deep pockets would oppose this law, those costs were assumed to be at the upper end of the range estimated by Downing (i.e., 7.1% of the annual lawsuit claims) (48).

#### 2.3.4 Calculation of benefit–cost ratios and the incremental cost-effective ratios

Benefit–cost ratios and incremental cost-effectiveness ratios were calculated based on the benefits of alcohol harm reduction interventions (i.e., cost savings from a reduction in resource use and the value of health improvement) and intervention costs using the formulas presented in Section 2.3.1.

## 3 Results

Measures used to assess the extent to which an intervention represents value for money were the benefit–cost ratio (BCR) and incremental cost-effectiveness ratio (ICER). A benefit–cost ratio greater than one indicates monetary benefits from implementing the intervention exceed monetary costs, with higher positive BCRs representing a better return (49). Interpreting the level at which the ICER represents value for money is less straightforward. One approach used to draw conclusions about an intervention’s cost-effectiveness is that of a threshold or reference value for the ICER, above which an intervention is not considered cost-effective and below which it would be considered cost-effective (50). While objections have been raised against the notion of an ICER threshold value, it is commonly used as a benchmark value against which to compare ICERs. While no official statement has been made about a threshold value for health services in Australia, an estimated willingness to pay threshold of A$50,000 (US$33,500) per QALY compared to the best alternative is commonly cited in Australian policy reviews (51).

Within each of the categories and types of alcohol harm prevention programs, several interventions were found to represent value for money (Tables 4, 5). The following interventions were found to be cost saving from a societal perspective:

- Availability of alcohol: enforce underage drinking laws (BCR = 46.0); enforce laws against serving intoxicated patrons (BCR = 35.0).
- Marketing of alcoholic beverages: alcohol advertising ban (BCR = 7.6).
- Pricing policies: Minimum price for alcohol of $0.95 per drink (BCR = 2.8); taxing wine at the same excise tax rate as low-alcohol beer instead of its wholesale sales price (BCR = 8.8), or taking all types of alcohol based on their alcohol content (cost-saving if one maintains the same total alcohol tax revenue, with extremely high benefit–cost ratios if the tax rate is chosen to maintain the current deadweight loss from taxation or is set at the spirits tax rate).
- Drink driving policies and countermeasures: law to allow administrative license revocation based on breath testing (10.3); administrative license revocation (BCR = 8.2); zero alcohol tolerance for drivers under 18 years (BCS = 7.2); alcohol testing ignition interlock mandated for all offenders (BCR = 6.1); raising RBT rate from 0.5 to 1.0/driver/year (BCR = 5.5); mass media campaigns to reduce drink driving (BCR = 4.7).
- Workplace interventions: Prime for Life (BCR = 18.2); Team Resilience (BCR = 10.6).
- Health sector programs: Screening for heavy drinking in a hospital inpatient setting (BCR = 29.0); screening for heavy drinking in primary care setting (BCR = 12.2).
- Youth development programs: Strengthening Families program (BCR = 21.1); Caring School Community program (BCR = 10.3).
- School-based substance abuse prevention: All Stars (BCR = 45.3); Family Matters (BCR = 31.9); Positive Action (BCR = 20.7).

**Table 4:** Cost-effectiveness of environmental interventions, workplace interventions and health sector programs.

Interventions	Cost	Cost units (per)	Resource savings	Intangible savings	Total Savings	BCR	Cost/QALY
Availability of alcohol							
Enforce laws against serving intoxicated patrons	$1	driver	$8	$27	$35	35.0	Net saving
Raise minimum legal drinking age to 19 years	$401	youth age 19	$142	$679	$821	2.0	$69,355
Mandatory server training to deny service to intoxicated and underage patrons	$100	driver	$37	$185	$222	2.2	$62,231
Licensed establishment liability for harm caused by over-servicing patrons	$6	adult	$5	$20	$25	4.2	$8,788
Enforce underage drinking laws	$4	youth ages 12–18	$31	$153	$184	46.0	Net saving
10% alcohol outlet density reduction	$3,095	M population	$2,410	$13,626	$16,036	5.2	$9,151
10% fewer sales hours/week	$7,792	M population	$6,026	$34,064	$40,090	5.1	$9,432
Marketing of alcoholic beverages							
Alcohol advertising ban	$1,329	M population	$2,167	$7,976	$10,144	7.6	Net saving
TV alcohol advertising ban	$12,372	M population	$9,643	$54,501	$64,144	5.2	$9,114
Pricing policies							
Minimum price for alcohol of A$0.95 per drink	$10	drinker/year	$12	$16	$28	2.8	Net saving
Volumetric tax on alcohol, same total tax revenue	-$10	10,000 drinks	$1	$6	$7	No cost	Net saving
Volumetric tax on alcohol, same deadweight loss	$0.04	10,000 drinks	$11	$51	$61	1,537	Net saving
Volumetric tax on alcohol at spirits tax rate	$151	10,000 drinks	$1,651	$7,913	$9,564	63	Net saving
Replace price-based wine tax with excise tax at beer rate	$3	10,000 drinks	$5	$22	$27	8.8	Net saving
Drink driving policies and countermeasures							
Administrative license revocation	$4,330	license revoked	$5,265	$30,105	$35,370	8.2	Net saving
Law to allow administrative license revocation based on breath testing	$4,065	license revoked	$6,269	$35,797	$42,066	10.3	Net saving
Alcohol-testing ignition interlock permitted	$1,912	vehicle equipped	$739	$4,041	$4,780	2.5	$52,855
Raise random breath test rate from 0.5 to 1/driver/year	$21	driver tested	$22	$91	$113	5.5	Net saving
Zero alcohol tolerance for drivers under 18 years	$78	driver	$93	$471	$564	7.2	Net saving
Mass media campaign to reduce drink driving	$5,389	1,000 population	$5,446	$20,056	$25,502	4.7	Net saving
Saturation patrols plus media campaigns	$51,406	10,000 drivers	$40,487	$167,137	$207,624	4.0	$11,890
Vehicle impoundment for drink driving offenses	$1,648	impoundment	$603	$2,646	$3,248	2.0	$71,874
Electronic house arrest for drink driving offenses	$2,868	house arrest	$1,960	$1,619	$3,579	1.2	$102,161
Intensive probation for drink driving offenses plus treatment	$1,511	probation	$1,075	$2,506	$3,581	2.4	$31,684
Driving under the influence court	$3,092	client	$163	$6,846	$7,009	2.3	$77,853
Alcohol testing ignition interlock mandated for all offenders	$1,912	vehicle equipped	$1,794	$9,814	$11,608	6.1	Net saving
Workplace interventions							
Prime for Life employer-sponsored web-based health promotion program that includes substance abuse prevention	$19	participating worker	$55	$299	$354	18.2	Net saving
Team Awareness program to promote group cohesiveness, stress coping, and peer support that includes substance abuse prevention (retail and restaurant workers)	$268	participating worker	$228	$1,245	$1,473	5.5	$5,828
Team Resilience: an adaptation of Team Awareness for workers under age 26 years	$269	participating worker	$445	$2,421	$2,865	10.6	Net Saving
PREVENT: a facilitated set of employee discussions in small groups focused on recognising the need for and planning changes in substance use and financial management for young workers	$494	worker participating	$209	$1,180	$1,389	2.8	$43,918
Health sector programs							
Screening for heavy drinking in primary care setting and brief motivational intervention when indicated	$353	person treated	$439	$3,872	$4,311	12.2	Net saving
Screening for heavy drinking in hospital inpatient setting and brief motivational intervention when indicated	$129	person treated	$382	$3,376	$3,758	29.0	Net saving
Screening for heavy drinking in emergency department care setting and brief motivational intervention when indicated	$350	person treated	$313	$2,761	$3,074	8.8	$2,460

**Table 5:** Cost-effectiveness of youth development and school-based substance abuse prevention programs.

	Cost/ student	Resource savings	Intangible savings	Total savings	BCR	Cost/QALY
Youth Development Programs						
Adolescent Transitions: a parenting skills program combined with universal, indicated, and selective prevention, ages 10–18	$2,035	$456	$2,804	$3,260	1.6	$134,413
Across Ages: a program to strengthen adult and youth bonds through mentoring, community service and family activities, ages 9–13	$2,930	$503	$3,008	$3,511	1.2	$194,624
Social Competence Promotion: a program to enhance social competence to reduce the use of violence and improve conflict resolution, ages 11–14	$593	$511	$3,140	$3,651	6.2	$6,232
Communities That Care: needs assessment, followed by implementation of evidence-based youth interventions	$819	$195	$3,598	$3,793	4.6	$40,145
Strengthening Families Program, parent–child behavioral training designed to prevent substance use, ages 12–13	$1,479	$1,783	$29,469	$31,252	21.1	Net Savings
Caring School Community: program to build sense of school community, ages 9–13	$388	$643	$3,350	$3,993	10.3	Net Savings
Good Behavior Game, classroom management strategy to teach youth to comply with rules, ages 6–9	$102	$46	$3,157	$3,203	31.4	$4,064
Guiding Good Choices (Preparing for Drugfree Years), parent–child behavioral training, ages 12–13	$1,194	$999	$4,325	$5,324	4.5	$10,830
School-Based Substance Abuse Prevention						
Family Matters: a family-focused program to reduce tobacco and alcohol use, ages 12–14	$265	$412	$8,039	$8,451	31.9	Net Savings
All Stars: decision-making, goal setting, and peer pressure resistance skills training, ages 11–14	$236	$554	$10,146	$10,700	45.3	Net Savings
Life Skills Training, 3-year program, ages 13–16	$375	$241	$4,591	$4,832	12.9	$6,756
Positive Action: a school-wide positive behavior program aimed at improving social and emotional learning and school climate, grades 3–8	$1,969	$3,545	$37,310	$40,855	20.7	Net Savings
Positive Action as above, grades 3–5	$1,063	$936	$12,765	$13,701	12.9	$2,321
Project Northland: school-based child–parent training, 3-years program, ages 12–15	$670	$328	$9,003	$9,331	13.9	$8,754
Project STAR: a school/family/community/media to prevent drug and alcohol use, 2-year program, ages 12–15	$670	$227	$5,640	$5,867	8.8	$18,195
Project Toward No Drugs, ages 15–19 yrs. (high school)	$303	$308	$1,218	$1,526	5.0	$0
Too Good for Drugs, school-based life skills, ages 12–14	$139	$98	$583	$681	4.9	$16,934

## 4 Discussion

This study estimates the cost–benefit and cost per QALY saved for 49 alcohol misuse prevention and harm reduction strategies. From an economic standpoint, promising interventions were identified across all categories; however, the expected return on investment should be interpreted in the light of the quality of underlying studies. For example, while all pricing policies were found to generate net savings, only the intervention ‘replacing price-based wine tax with excise tax at beer rate’ received an “A” quality rating. Additionally, the analysis adopted a societal perspective, meaning that the calculated return on investment would differ from narrower perspectives such as government, heavy drinkers, or the alcohol industry.

The benefit–cost estimates related to alcohol tax interventions rely on published estimates of the substitution between alcoholic beverages based on relative prices; however, these studies considered smaller price changes than those proposed here. As a result, the estimated costs to consumers may be understated. Nevertheless, increased taxation appears to offer an excellent return on societal investment.

Other reviews of the cost-effectiveness of alcohol harm reduction policies report broadly similar findings. In their rapid evidence review, Burton et al. focused on alcohol control policies from an England and Wales perspective (52) with support found for cost-effectiveness of measures addressing affordability (i.e., pricing and tax policies), marketing and availability. Similarly, a rapid synthesis by Guindon et al. (53) and a recent systematic review of economic evaluations of interventions to prevent alcohol use by Le et al. (54) had similar findings, with Le et al. adding selective screening with or without brief interventions for at-risk adults and some school-based interventions combined with parent/carer interventions to the list of cost-effective interventions.

While our analysis draws on studies from high income countries, the findings are relevant to LMIC settings. The SAFER initiative launched in 2018 by the World Health Organization and international partners was established to provide support for member states in reducing the harmful use of alcohol (55). Its focus was on the most cost-effective priority interventions (“best buys”) to prevent and reduce alcohol-related harm across each of the following: strengthen restrictions on alcohol availability; advance and enforce drink driving countermeasures; facilitate access to screening, brief interventions, and treatment; enforce bans or comprehensive restrictions on alcohol advertising, sponsorship, and promotion; and raise prices on alcohol through excise taxes and pricing policies. Specific interventions within these categories have been examined to provide comparative value for money estimates to use in prioritizing interventions for funding.

A strength of this study lies in producing a consistent set of cost-effectiveness estimates across and within each broad category of interventions, achieved by adjusting published data for comparability. Most other reviews report findings without standardizing cost-effectiveness estimates across studies. Covering a wide range of policies, the study offers an evidence base to guide decision on interventions most likely to be both technically efficient (i.e., which drink driving policy is likely to be most cost-effective) and allocatively efficient (i.e., what package of alcohol harm reduction provides best return on investment) (56). Presenting consistently calculated return on investment for a broad range of interventions facilitates policy debate and priority-setting.

However, limitations include the age and variable quality of some data on intervention costs and effectiveness, and the sensitivity of benefit–cost ratios to the value of statistical life used to monetize QALY losses. Moreover, the 3% discount rate applied to future cost savings aligns with international standards (57),but might be higher than the typical differential between inflation and the return on safe investments. Lack of data on standard errors, especially for intervention costs, precluded calculating confidence intervals around the estimates.

Future research should focus on refining these estimates by incorporating emerging data, especially from low- and middle-income countries. Furthermore, expanding the analysis to include long-term societal and economic impacts, as well as equity considerations, would offer a more comprehensive understanding of intervention benefits. Developing dynamic models that account for changing social, economic, and policy contexts could also bolster the robustness and relevance of future evaluations, ultimately strengthening evidence-based decision-making in alcohol harm reduction.

## 5 Conclusion

Economic evaluation enables policy and program managers to make informed resource allocation decisions for alcohol harm reduction. This study offers the most comprehensive estimates of the costs and benefits associated with various prevention strategies, drawing on recent published studies.

Harmful and hazardous alcohol consumption results in a wide range of adverse consequences, which can be mitigated through effective prevention programs and strategies. Given the diversity of proven interventions, optimal resource allocation requires selecting complementary, politically feasible, and culturally and demographically relevant measures to maximize return on investment within available funding. Developing a coherent package that complements existing interventions is of particular importance (13).

To translate these findings into effective action, policymakers should prioritize implementing a combination of interventions targeting multiple aspects of alcohol-related harm. For example, combining measures that prevent underage drinking with those targeting repeat impaired drivers can amplify overall impact (58).

A structured decision-making approach can improve practical application. Policymakers should first use the cost-effectiveness estimates to eliminate interventions with questionable return on investment. Then, considerations such as political feasibility, local priorities, appropriateness for the target population, cultural sensitivity, affordability, and impact timing (weeks versus years) should guide final selection. Political feasibility is especially important. A slightly less cost-beneficial program can be superior if the alternative with the higher return has a lower chance of widespread implementation or involves a long delay in implementation. All things are not equal when selecting a package that yields the maximum gains at the lowest possible price. Other factors, such as aggregate benefits obtained, overlapping effects, spillover costs and benefits (e.g., a youth alcohol misuse prevention program that also reduces tobacco use), equity, and government cost can outweigh the gain differential. For example, alcohol pricing interventions evaluated here tend to be inequitable, disproportionately affecting low-income drinkers. Taxation changes can generate substantial government revenues, but also could face a political quagmire (13).

In practical terms, the insights from this research can guide the prioritization of interventions that are not only cost-effective but also feasible and culturally sensitive. By aligning economic evidence with contextual realities, policymakers and stakeholders can develop a comprehensive package of alcohol harm reduction strategies that deliver tangible benefits in diverse settings.

## Data Availability

All data inputs are referenced in the article. The only data input not referenced are the unit costs in the Supplementary material. The report from which these data are drawn can be obtained from the corresponding author.
