# Supplementary material for: Economic evaluation of evidence-based strategies to reduce unhealthy alcohol use: a resource allocation guide
Source: Front Public Health. 2025 Aug 11;13:1466552. doi: 10.3389/fpubh.2025.1466552 (PMC12376196; doi:10.3389/fpubh.2025.1466552)
Supplement: Supplementary file 1 [file Table_1.docx]

**SUPPLEMENTARY TABLE**

**Per person and per event cost of attributable harm**

| **Alcohol harm (cost) ratios** | **A$ (2018)** |
| --- | --- |
| Mean cost per person | 2,833 |
| Mean cost per adult population | 3,676 |
| Mean cost per population 12 and over | 3,360 |
| Mean cost per person who consumes alcohol | 4,309 |
| Mean cost per underage drinker | 4,309 |
| Mean cost per standard drink of alcohol consumed | 4.02 |
| Mean cost per crash | 47,646 |

Cost data was obtained from the following report, which is available upon request. Denominators were obtained from official published data.

Hendrie D, Miller TR, Randall S, Brameld K and Chikritzhs, T. Costs of alcohol-related harm in Western Australia. Report prepared for Healthway. Perth: School of Public Health Curtin University; 2018.
